# Supplementary material for: Beta 2-Adrenergic Receptor in Circulating Cancer-Associated Cells Predicts for Increases in Stromal Macrophages in Circulation and Patient Survival in Metastatic Breast Cancer
Source: Int J Mol Sci. 2022 Jun 30;23(13):7299. doi: 10.3390/ijms23137299 (PMC9266803; doi:10.3390/ijms23137299)
Supplement: Supplementary file 1 [file ijms-23-07299-s001.zip › ijms-1765507-supplementary.pdf]

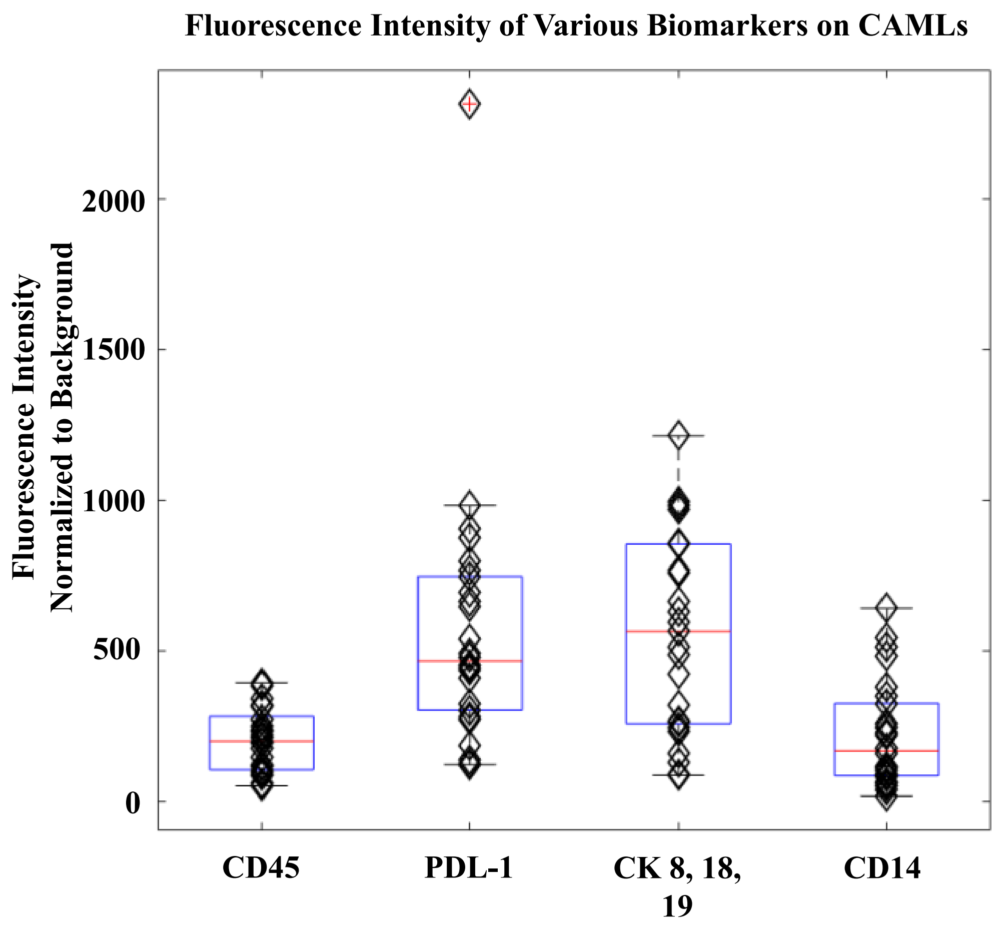

Supplemental Figure S1: Fluorescence Intensity of various Biomarkers on CAMLs. a. Whisker Plots of intensities of CD45, PDL-1, CK 8, 18, 19, and CD14 found in CAMLs of the Breast Cancer patients stained with B2AR.

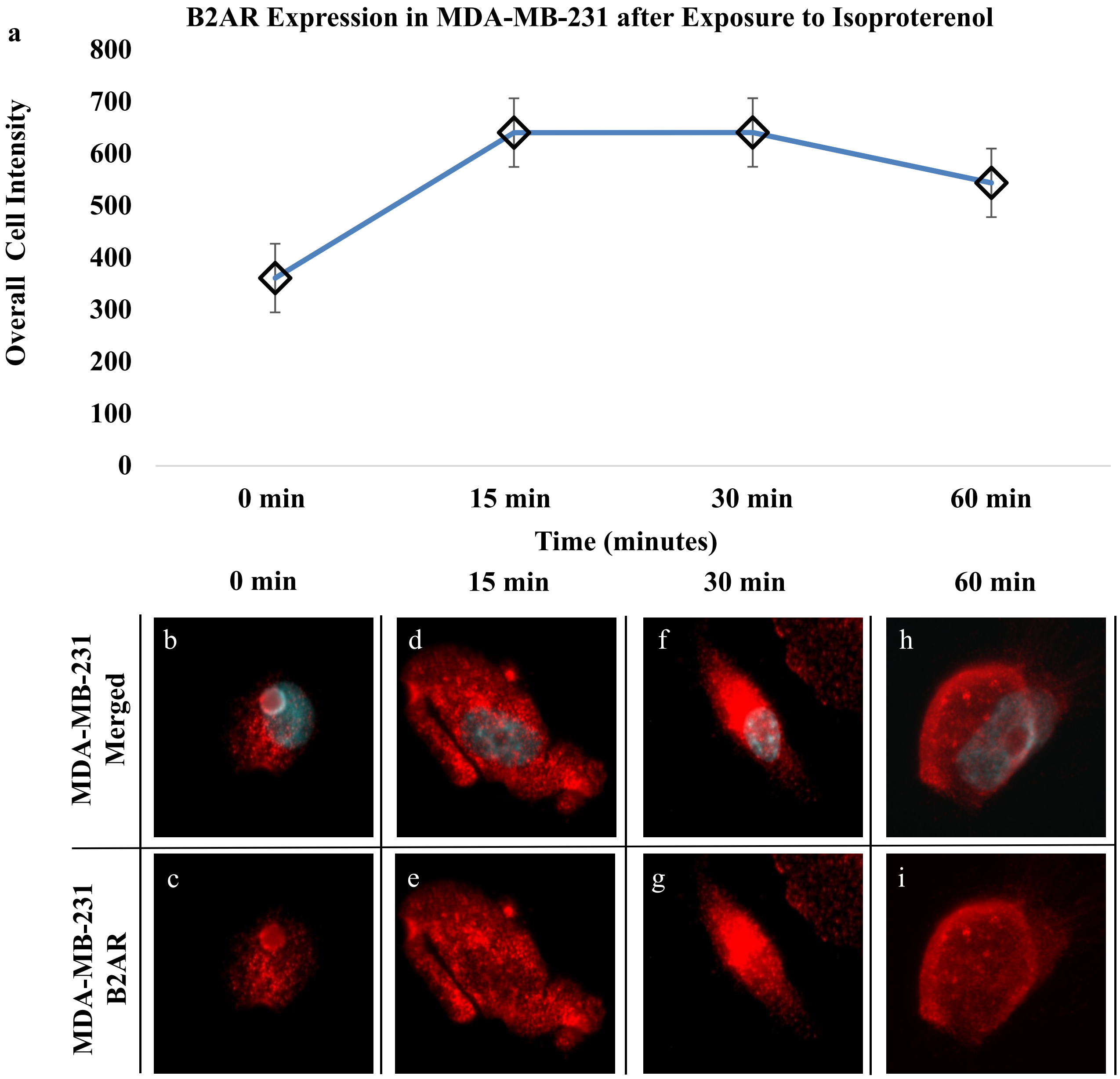

Supplemental Figure S2: B2AR intensity changes, internalization and upregulation after exposure to isoproterenol: a. Bioassay intensity of B2AR in MDA-MB-231 cell line compared to exposure times using 2 µm isoproterenol. Error bars=S.E**. b & c.** Inactive B2AR is localized to the perimeter of the cell surface. **d & e**. After activation B2AR internalizes into endocytic compartments within the cells, causing a lowering of overall signal intensity. **f & g** After 30 min the B2AR can be seen within endocytic compartments and on the cell surface, as well as increasing in signal as B2AR begins recycling to the surface. **h & i**. Overall cell increases B2AR production in response to high stimuli. **b, d, f & h.** Merged cells of DAPI (blue), cytoplasm stain (green), and B2AR (red)

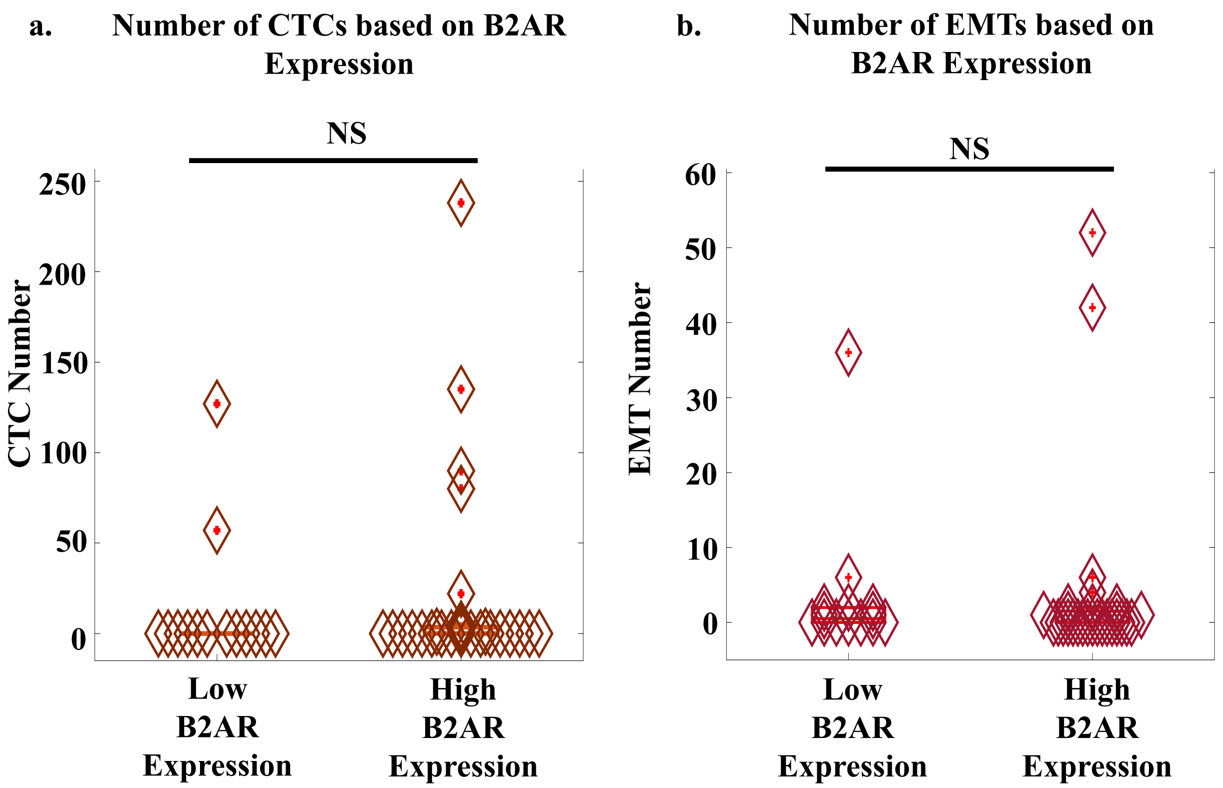

Supplemental Figure S3: Relationship of B2AR Expression on CAMLs and number of CTCs and EMTs in circulation. a. Whisker Plots of patient's CTCs number based on high/low expression of B2AR. Wilcoxon ranked sum (*p=0.1565). Red circles=data outliers. **b.** Whisker Plots of patient's EMTs based on high/low expression of B2AR. Wilcoxon ranked sum (**p=0.5527).
